# Preparation and Characterization of Highly Conductive PVDF/PAN Conjugate Electrospun Fibrous Membranes with Embedded Silver Nanoparticles

**DOI:** 10.3390/polym16243540

**Published:** 2024-12-19

**Authors:** Siyang Wu, Luyu Zhang, Xiaochun Qiu, Yuntai Guo, Liangliang Dong, Mingzhuo Guo, Jiale Zhao

**Affiliations:** 1 College of Engineering and Technology, Jilin Agricultural University, Changchun 130118, China; wsy@jlau.edu.cn (S.W.); qiuxiaochun0131@163.com (X.Q.); guoyuntai@yeah.net (Y.G.); liangliangdong@163.com (L.D.); 2 College of Biological and Agricultural Engineering, Jilin University, Changchun 130022, China; lyzhang@jlu.edu.cn

**Keywords:** conjugate electrospinning, silver nanoparticles, conductive fibrous membrane, wearable sensor

## Abstract

This study reports the development of highly conductive and stretchable fibrous membranes based on PVDF/PAN conjugate electrospinning with embedded silver nanoparticles (AgNPs) for wearable sensing applications. The fabrication process integrated conjugate electrospinning of PVDF/PAN, selective dissolution of polyvinylpyrrolidone (PVP) to create porous networks, and uniform AgNP incorporation via adsorption-reduction. Systematic optimization revealed that 10 wt.% PVP content and 1.2 mol/L AgNO_3_ concentration yielded membranes with superior electrical conductivity (874.93 S/m) and mechanical strength (2.34 MPa). The membranes demonstrated excellent strain sensing performance with a gauge factor of 12.64 within 0–30% strain and location-specific sensing capabilities: moderate movements at wrist (Δ*R*/*R*_0_: 98.90–287.25%), elbow (124.65–300.24%), and fingers (177.01–483.20%) generated stable signals, while knee articulation exhibited higher sensitivity (459.60–1316.48%) but significant signal fluctuations. These results demonstrate the potential of the developed conductive porous PVDF/PAN composite fibrous membranes for applications in wearable sensors, flexible electronics, and human-machine interfaces, particularly in scenarios requiring moderate-range motion detection with high reliability and stability. The findings suggest promising opportunities for developing next-generation wearable sensing devices through the optimization of conjugate electrospun fibrous membranes.

## 1. Introduction

Flexible and wearable electronic devices have emerged as a significant research focus due to their extensive applications in health monitoring systems, human-machine interfaces, and smart textiles [1,2,3,4,5]. These devices enable seamless integration of sensing capabilities into daily activities, facilitating continuous monitoring of physiological signals, body movements, and environmental conditions. However, several critical challenges remain to be addressed for the practical implementation of wearable electronics. A fundamental requirement involves the development of sensing materials that simultaneously exhibit superior electrical conductivity while maintaining mechanical flexibility, stretchability, and user comfort [6,7].

Among diverse materials investigated for wearable sensors, polymer-based nanofibers have demonstrated significant potential, attributed to their distinctive characteristics, including high porosity, large surface area, and tunable mechanical properties [8,9,10]. Electrospun nanofibers, in particular, have garnered substantial research attention due to their facile fabrication process, controllable morphology, and capability for functionalization with conductive nanomaterials [11,12]. Currently, various conductive fillers have been extensively investigated for flexible sensors, including carbon nanotubes (CNTs), graphene, conductive polymers, and metallic nanoparticles [6,8]. While each material offers distinct advantages, silver nanoparticles demonstrate superior electrical conductivity among metallic nanoparticles. Moreover, the chemical reduction method for AgNP synthesis provides precise control over particle size and distribution, enabling optimal integration within polymer matrices. These characteristics, combined with their excellent chemical stability and established processing methods, make AgNPs particularly attractive for developing high-performance conductive membranes. The incorporation of AgNPs through controlled chemical reduction presents opportunities for achieving enhanced conductivity and sensing performance while maintaining the structural integrity of electrospun fibers.

The incorporation of conductive fillers, such as silver nanoparticles (AgNPs), into electrospun nanofibers has been shown to significantly enhance their electrical and sensing performance [13,14]. AgNPs are widely utilized due to their high electrical conductivity, antibacterial properties, and facile synthesis methods [15]. The size and surface chemistry of AgNPs play crucial roles in determining their performance and stability. In a notable study, Haidari et al. [16] demonstrated that ultrasmall AgNPs (<3 nm) exhibit superior performance when properly stabilized in polymer matrices. Their work showed that optimized surface modification and size control not only enhance AgNP’s stability but also significantly improve their functional properties. Physical mixing, if not properly controlled, may lead to AgNP agglomeration, which could affect the uniformity of particle distribution and compromise the overall performance of the composite materials. However, with appropriate process parameter control and surface modification, physical mixing can achieve effective AgNP dispersion.

Conventional methods for incorporating AgNPs into polymer fibers, such as direct blending and surface coating, often face several challenges. Direct blending of AgNPs with polymer solutions before electrospinning typically results in nanoparticle aggregation and uneven distribution, leading to inconsistent electrical properties [17,18]. Surface coating methods, while simple to implement, frequently suffer from poor adhesion between AgNPs and fiber surfaces, resulting in particle detachment during mechanical deformation [19]. Additionally, post-processing methods like thermal reduction or UV irradiation can potentially damage the polymer fiber structure and limit the achievable conductivity [20,21]. In contrast, our in-situ reduction approach enables uniform AgNP distribution throughout the porous PVDF network while maintaining strong particle-polymer interfacial adhesion, thereby ensuring stable electrical performance under mechanical strain. To overcome these technical challenges, conjugate electrospinning represents an advanced methodology for fabricating composite nanofibers with enhanced properties. This approach involves the simultaneous spinning of multiple polymers through specialized nozzles, enabling the formation of core-sheath, side-by-side, or island-in-the-sea fiber architectures [22,23,24]. This methodology facilitates strategic polymer combination, leveraging individual material properties to generate synergistic effects. Through careful polymer selection and process parameter optimization, including applied voltage, polymer flow rate, solution concentration and tip-to-collector distance [25], conjugate electrospinning enables precise control over functional material distribution within the nanofiber matrix [26,27,28].

Polyvinylidene fluoride (PVDF) and polyacrylonitrile (PAN) represent widely utilized polymers in electrospinning applications, each offering distinct advantages. PVDF exhibits exceptional thermal stability, chemical resistance, and mechanical properties [29,30], while PAN provides superior mechanical strength and serves as an effective platform for metal nanoparticle immobilization due to its nitrile group functionality [31,32]. The conjugate electrospinning of PVDF and PAN potentially yields nanofibers with enhanced properties, combining the advantageous characteristics of both polymers.

The present study describes a novel approach for fabricating highly conductive and stretchable PVDF/PAN conjugate electrospun fibrous membranes with embedded AgNPs. Recent studies in conductive and stretchable membranes often utilize single polymer matrices, which result in limited mechanical properties. In contrast, our primary innovation lies in the strategic integration of a dual-fiber system and efficient AgNPs incorporation method. Initially, conjugate electrospinning methodology is implemented to produce composite nanofibers with an interconnected network structure, utilizing the complementary properties of PVDF and PAN. Unlike conventional single-component systems, the PAN fibers contribute mechanical strength and stretchability to the composite membrane, while PVDF fibers function as templates for silver ion adsorption and reduction processes. Furthermore, while traditional surface coating approaches for conductive materials often face challenges in achieving stable attachment, our study develops an efficient in-situ reduction method. To optimize conductivity and sensing capabilities, a porous structure is introduced into the PVDF fibers through selective polyvinylpyrrolidone (PVP) dissolution. This porous PVDF network serves as an effective scaffold for in-situ reduction and immobilization of AgNPs, ensuring strong interfacial adhesion and facilitating high loading and uniform distribution of AgNPs within the fibrous matrix. Through systematic optimization of PVDF/PAN ratios and processing parameters, our approach achieves an effective balance between electrical conductivity (874.93 S/m) and mechanical properties (2.34 MPa). The synergistic integration of conjugate PVDF/PAN nanofibers and AgNPs-loaded porous PVDF network substantially enhances the electrical, mechanical, and sensing properties of the resulting fibrous membranes. Systematic characterization studies of the AgNPs-loaded PVDF/PAN fibrous membranes elucidate structure-property relationships. The investigation encompasses a detailed analysis of AgNP loading effects on electrical conductivity, mechanical strength, and strain-sensing performance. The practical applicability of the prepared membranes is validated through integration into wearable sensors for human motion detection.

This research presents significant advances in the development of highly conductive and sensitive fibrous membranes applicable to wearable sensors and flexible electronics. These results advance the field of functional materials for smart textiles and human-machine interfaces, establishing new paradigms for the practical implementation of electrospun conductive fibers in health monitoring and motion detection applications. This investigation elucidates critical design principles and fabrication methodologies for high-performance, multifunctional fibrous membranes suitable for next-generation wearable sensing devices.

## 2. Materials and Methods

### 2.1. Materials

Polyvinylidene fluoride (PVDF, Mw ~534,000, PDI 1.8) and polyacrylonitrile (PAN, Mw ~150,000, PDI 2.1) were obtained from Sigma-Aldrich (St. Louis, MO, USA). N,N-dimethylformamide (DMF, ≥99.8%) and acetone (≥99.9%) were sourced from Aladdin Reagent (Shanghai, China). Polyvinylpyrrolidone (PVP, K30, Mw ~40,000, PDI 1.6) was procured from Macklin Biochemical (Shanghai, China). Silver nitrate (AgNO_3_, ≥99.8%) and L-ascorbic acid (AA, ≥99.7%) were supplied by Sinopharm Chemical Reagent (Shanghai, China). All reagents were of analytical grade and utilized without further modification. Deionized water with a resistivity of 18.2 MΩ·cm was produced from a Milli-Q system and used throughout the experiments.

### 2.2. Preparation of Conductive Porous PVDF/PAN Composite Fibrous Membrane

#### 2.2.1. Preparation of PVDF/PVP/PAN Composite Fibrous Membrane

The fabrication of PVDF/PVP/PAN composite fibrous membrane involved conjugate electrospinning. Initial preparation involved forming a homogeneous precursor solution by dissolving PVDF (1.554 g) in a mixed solvent of DMF and acetone (6:4 *v*/*v*) under magnetic stirring at 500 rpm for 2 h at 50 °C. To investigate the influence of PVP content on the porosity and mechanical properties of the resulting porous PVDF membrane, PVP was added to the PVDF solution at various concentrations (5, 10, and 15 wt.%). Separately, a PAN solution was prepared by dissolving PAN in DMF.

The PVDF/PVP and PAN solutions were loaded into two separate 5-mL syringes and electrospun simultaneously using a commercial electrospinning device (HZ−12, Qingdao Nuokang Environmental Protection Technology Co., Ltd., Qingdao, China) equipped with a conjugate spinneret. The flow rate was controlled by the syringe pump advancing speed, with a default speed of 0.8 mm/min (corresponding to approximately 0.023 mL/min) for the 1:1 PAN:PVDF ratio, and proportionally adjusted to achieve the other ratios (0.5:1 and 1.5:1). These ratios were specifically selected to investigate the membrane properties under different component dominance: PAN-dominant (0.5:1), balanced composition (1:1), and PVDF-dominant (1.5:1). The electrospinning process was conducted in a controlled environment chamber with integrated dehumidification and heating modules, maintaining the relative humidity at 45 ± 5% and temperature at 25 ± 2 °C to ensure consistent fiber formation and morphology. The applied voltages were +9 kV and −9 kV, and the receiving distance was 12 cm. The PVDF/PVP/PAN composite fibrous membrane was collected on a rotating drum collector at 80 rpm [33].

#### 2.2.2. Preparation of Conductive Porous PVDF/PAN Composite Fibrous Membrane via PVP Removal and AgNPs In-Situ Reduction

The porous structure within PVDF fibers was generated through the selective dissolution of water-soluble PVP by immersing the as-spun PVDF/PVP/PAN membrane in deionized water for 3 h. During this process, the PVP was dissolved and leached out from the fibers, leaving behind a porous PVDF fibrous network interlaced with PAN fibers. The resulting porous PVDF/PAN composite membrane was then dried at room temperature. Additionally, to enhance the removal of PVP, the composite membrane was subjected to ultrasonic treatment in deionized water for 30 min.

**Figure 1:**
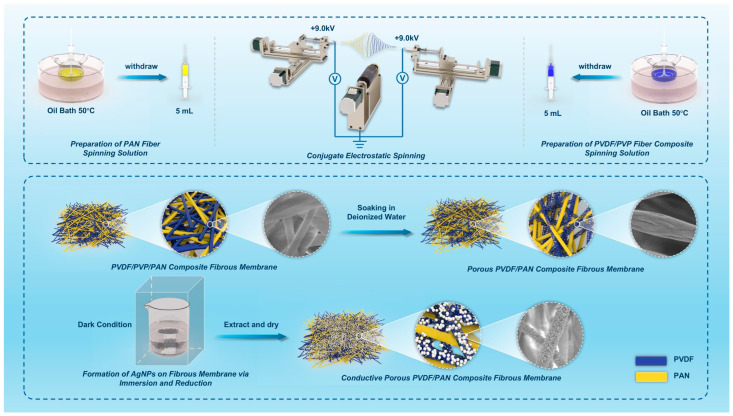
Schematic illustration of the fabrication process for the conductive porous PVDF/PAN composite fibrous membrane.

The conductive porous PVDF/PAN composite fibrous membrane was fabricated through an adsorption-reduction approach. Firstly, the porous PVDF/PAN membrane was immersed in an AgNO_3_/PVP mixed solution for 6 h under dark conditions to allow the adsorption of Ag^+^ ions into the porous PVDF network. The concentrations of AgNO_3_ solution used were 0.2, 0.4, 0.6, 0.8, 1.0, 1.2, and 1.4 mol/L. Subsequently, the Ag^+^-loaded membrane was transferred to an ascorbic acid/PVP reduction solution and soaked for another 20 h in the dark. During this step, the adsorbed Ag^+^ ions were reduced to AgNPs, which were anchored within the porous PVDF matrix. Finally, the conductive porous PVDF/PAN composite fibrous membrane was thoroughly rinsed with deionized water to remove any residual reagents and then dried at room temperature. The overall fabrication process is schematically illustrated in Figure 1.

### 2.3. Characterization and Measurements

#### 2.3.1. Morphological Analysis

Morphological characterization was performed using scanning electron microscopy (SEM, EVO-18, Zeiss, Oberkochen, Germany) operating at an acceleration voltage of 15 kV. The investigation focused on two aspects: (i) the effect of varying PVP contents (5, 10, and 15 wt.%) on the porous structure formation in PVDF/PAN composite membranes, and (ii) the morphological characteristics of the conductive porous PVDF/PAN composite fibrous membrane after AgNPs incorporation. Prior to imaging, all samples underwent sputter coating with a thin gold layer to enhance conductivity. The optimization of PVP content was determined through a comprehensive analysis of the resulting membrane morphology and pore structure characteristics.

#### 2.3.2. Fourier Transform Infrared Spectrum (FTIR) Analysis

Fourier transform infrared spectroscopy (FTIR, Equinox 55, Bruker, Karlsruhe, Germany) was employed to monitor the effectiveness of PVP removal from the PVDF/PAN composite fibrous membranes. The analysis was performed in attenuated total reflection (ATR) mode, comparing spectra before and after the water immersion process. Measurements were conducted in the wavenumber range of 500–4000 cm^−1^ (resolution: 4 cm^−1^, accumulation: 32 scans), with particular attention to the characteristic absorption bands of PVP. The disappearance of PVP-specific peaks served as an indicator for the complete removal of the sacrificial polymer component.

#### 2.3.3. Mechanical Strength Analysis

Mechanical properties were evaluated using a universal testing machine (ZQ-990B, Zhiqu, Dongguan, China) under ambient conditions. Tensile stress-strain measurements were conducted on rectangular specimens (50 mm × 20 mm × 0.5 mm) at a constant crosshead speed of 50 mm/min. The analysis encompassed the mechanical behavior of pristine PVDF/PAN composite membranes, porous PVDF/PAN membranes after PVP removal, and AgNPs-loaded porous PVDF/PAN composite membranes. Cyclic loading-unloading tests consisting of 10 consecutive cycles were performed to assess structural stability and residual strain measurements were recorded to evaluate recovery characteristics. All mechanical tests were conducted in triplicate to ensure data reliability.

#### 2.3.4. Porosity Analysis

The porosity of the composite membranes was calculated based on the volume ratio method. The apparent density of the membrane was determined using the mass and volume of the membrane samples, while the bulk density was calculated based on the polymer composition. Measurements were performed on samples with precisely measured dimensions, and triplicate tests were conducted to ensure data reliability [34]. The porosity (*P*) was calculated according to the following equation:

(1) $$P=(1-\frac{m}{\rho Sh})\times 100\%$$

where *P* is the membrane porosity, *m* is the mass (g), *S* is the area (cm^2^), *h* is the thickness (cm), and *ρ* is the average density (g/cm^3^) of the PVDF and PAN polymers.

#### 2.3.5. Electrical Conductivity Test

The electrical conductivity of the composite membranes was measured using a digital multimeter (17B MAX-01, Fluke, Everett, WA, USA) under ambient conditions. Rectangular specimens (50 mm × 20 mm × 0.5 mm) were connected to the multimeter via copper electrodes placed at both ends of the sample. The electrical conductivity (*σ*, *S/m*) was determined using the measured resistance and sample dimensions:

(2) $$\sigma =\frac{L}{RA}$$

where *L* is the test distance (m), *R* is the resistance (Ω), and *A* is the cross-sectional area (m^2^) of the membrane.

The strain-dependent electrical response of the composite membranes was investigated to evaluate their potential as flexible strain sensors. A higher GF value indicates greater sensitivity of the membrane to mechanical deformation, making it more suitable for strain-sensing applications. All measurements were conducted under ambient conditions at a constant strain rate of 50 mm/min. Specimens (50 mm × 20 mm × 0.5 mm) were subjected to tensile strain while simultaneously monitoring the electrical resistance using a precision benchtop digital multimeter (34465A, Keysight, El Segundo, CA, USA). Copper electrodes were securely attached to both ends of the sample to ensure stable electrical contact during deformation. The sensitivity of the membrane to mechanical strain was quantified by the gauge factor (GF), which represents the relative change in electrical resistance per unit strain:

(3) $$GF=\frac{\Delta R}{\varepsilon R_{0}}=\frac{R-R_{0}}{\varepsilon R_{0}}$$

where *R*_0_ is the initial resistance before deformation, *R* represents the real-time resistance under strain, and *ε* is the applied tensile strain.

#### 2.3.6. Electrical Sensing Function Test

The capability of the composite membranes to detect human physiological movements was evaluated through real-time resistance measurements. The membrane sensors were carefully positioned on four representative body locations: fingers, wrists, elbows, and knees. A precision benchtop digital multimeter (34465A, Keysight, USA) was employed to acquire the dynamic resistance signals generated during natural body movements. The electromechanical response was quantified by calculating the relative resistance change (Δ*R*/*R*_0_), where *R*_0_ represents the initial resistance, and Δ*R* denotes the resistance variation during motion. This analysis provided insights into the membrane’s effectiveness as a wearable motion sensor across different movement patterns.

## 3. Results and Discussion

### 3.1. Characteristics of Porous PVDF/PAN Composite Membrane

**Figure 2:**
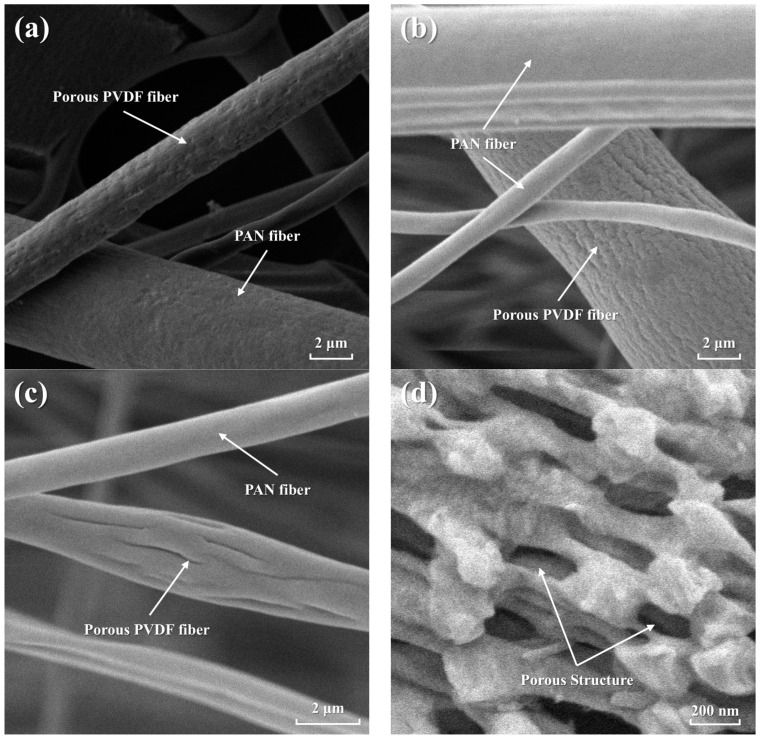
SEM microstructures of porous PVDF/PAN composite membranes with PVP contents of (**a**) 5 wt.%, (**b**) 10 wt.%, (**c**) 15 wt.%, and (**d**) high-magnification image of the membrane with 10 wt% PVP content showing detailed porous structure.

The influence of PVP content on porous PVDF/PAN composite membranes was systematically investigated through comprehensive characterization of microstructure morphology, porosity, mechanical properties, and FTIR analysis. The SEM micrographs depicted in Figure 2 illustrate the morphological characteristics of PVDF/PAN composite membranes fabricated with a volumetric flow ratio of 1:1 (PAN to PVDF), achieved through precise control of the electrospinning feed rates. This specific ratio was selected to ensure a balanced distribution of both fiber types within the observation field, facilitating comprehensive morphological analysis of both PAN and PVDF components under identical imaging conditions.

The morphological evolution of porous PVDF/PAN composite membranes as a function of PVP content (5, 10, and 15 wt.%) is illustrated in Figure 2a–c. To provide a more detailed visualization of the porous structure, high-magnification SEM imaging was conducted on the membrane with 10 wt% PVP content (Figure 2d). The higher resolution image clearly reveals well-defined pores within individual PVDF fibers, with pores exhibiting lengths ranging from 200 to 350 nm and widths of 80–200 nm. These distinct porous features, formed through the selective dissolution of PVP, create an interconnected network structure that serves as an effective template for subsequent AgNP incorporation. The uniform distribution and consistent size range of these nanoscale pores directly contribute to the enhanced surface area available for AgNP attachment while maintaining the structural integrity of the fibrous network. The incorporation of PVP into the PVDF matrix resulted in significant structural modifications, particularly evident in the surface morphology when compared to PAN fibers. While PAN fibers maintained their characteristic smooth surface topology, the selective dissolution of PVP by deionized water generated a hierarchical porous architecture within the PVDF component. The observed PAN fiber morphology and diameter distribution (2–3 μm) align well with previous reports by Gu et al. [35] and Khan et al. [36], who demonstrated similar fiber characteristics in core-sheath structured and graphene-embedded PAN systems, respectively. Similarly, the PVDF fiber dimensions (3–5 μm) fall within the range reported by Cozza et al. [37] and Mokhtari et al. [38] in their studies of electrospun PVDF-based piezoelectric systems, confirming the optimization of our electrospinning parameters. A direct correlation was observed between PVP content and pore development, with higher PVP concentrations yielding enhanced porosity. The fiber diameter distribution for both PVDF and PAN components predominantly ranged from 2 to 5 μm. This dimensional variation can be attributed to the inherent differences in rheological properties (viscosity), electrical characteristics (conductivity), and interfacial phenomena (surface tension) between the two polymers during the conjugate electrospinning process.

Previous investigations have established PVDF’s superior chemical stability and biocompatibility [33,39], making it more suitable for applications in filtration and separation technologies. Consequently, PVDF displayed superior adhesion when combined with PVP. The SEM images also reveal that PAN fibers were tightly intertwined with PVDF fibers, with PAN fibers providing support and an elastic skeleton for the composite membrane, ensuring the stability of PVDF fibers within the matrix. As demonstrated by Ince Yardimci’s et al. [40] comparative study, PAN and PVDF fibers exhibit distinct mechanical characteristics, with PAN showing superior tensile strength and Young’s modulus compared to PVDF. This inherent difference in mechanical properties provides a fundamental basis for our composite membrane design, where PAN fibers serve as the primary load-bearing component while PVDF fibers contribute to the functional aspects of the membrane. The integration of these complementary properties through conjugate electrospinning enables the development of membranes with enhanced mechanical stability and functional performance.

**Figure 3:**
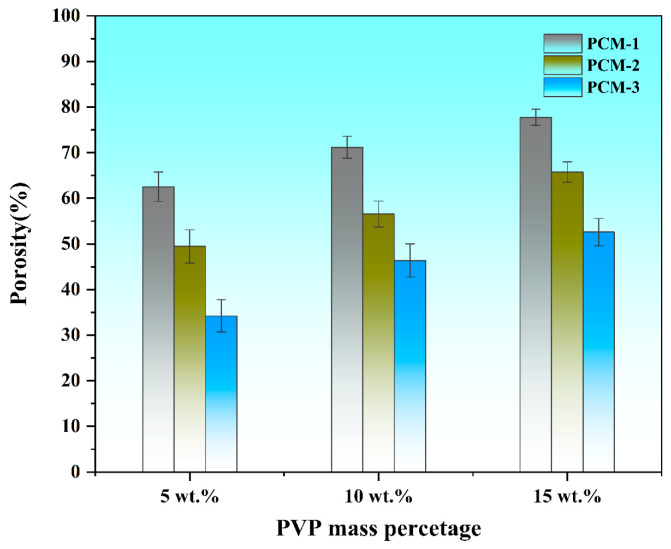
The porosity of porous PVDF/PAN composite membranes with different PVP contents.

The porosity characteristics of PVDF/PAN composite membranes were systematically investigated across varying PAN:PVDF ratios. Three distinct porous PVDF/PAN composite membranes (designated as PCM-1, PCM-2, and PCM-3) were fabricated through precise control of PAN and PVDF solution flow rates during electrospinning, resulting in PAN:PVDF content ratios of 0.5:1, 1:1, and 1.5:1, respectively. As shown in Figure 3, quantitative analysis revealed that membrane porosity exhibited a direct correlation with PVP content across all PAN:PVDF ratios. At a PAN:PVDF ratio of 0.5:1, the measured porosities were 62.52%, 71.17%, and 77.73% for PVP contents of 5 wt.%, 10 wt.%, and 15 wt.%, respectively. Similarly, at a PAN:PVDF ratio of 1:1, corresponding porosity values were 49.45%, 56.57%, and 65.73%. For the 1.5:1 ratio, porosities of 34.22%, 46.39%, and 52.59% were observed. The inverse relationship between porosity and PAN content can be attributed to the non-porous nature of PAN fibers, which remained structurally unmodified during the pore-formation process. These findings, corroborated by SEM morphological analyses, demonstrate that PVP content acts as the primary determinant in pore formation and structural development of the PVDF fibrous network. The direct correlation between PVP concentration and resultant porosity establishes PVP as the critical factor in controlling the microporous architecture of the composite membranes, confirming its essential role as a pore-forming agent in the PVDF/PAN system.

**Figure 4:**
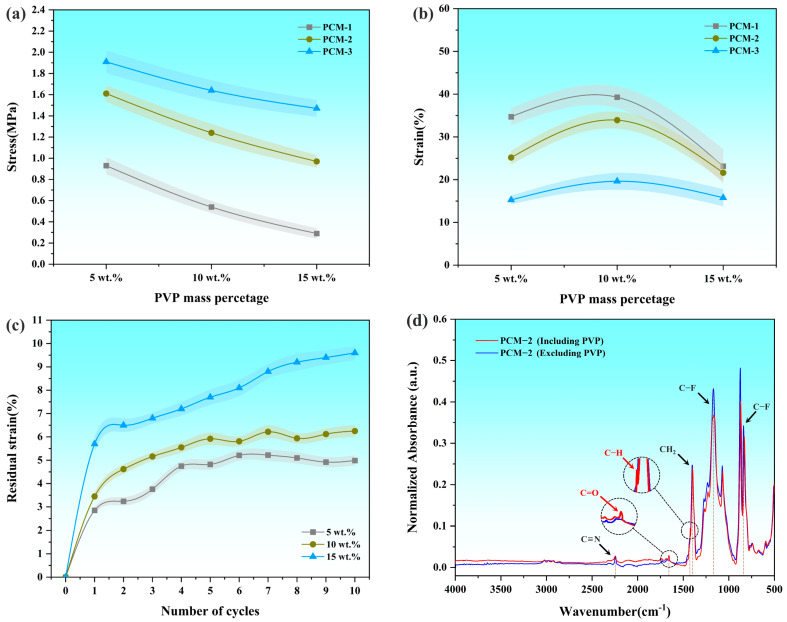
(**a**) Stress and (**b**) strain values of porous PVDF/PAN composite membranes with different PVP mass fractions, (**c**) residual strain of PCM-2 with 5 wt.%, 10 wt.% and 15 wt.% PVP contents, (**d**) Overlaid FTIR spectra of PVDF/PVP/PAN composite membrane before and after PVP dissolution.

The mechanical properties of porous PVDF/PAN composite membranes were systematically evaluated as a function of PVP content, as illustrated in Figure 4a,b. All three membrane variants (PCM-1, PCM-2, and PCM-3) demonstrated consistent patterns in their stress-strain behavior, where stress values exhibited an inverse relationship with PVP content, while strain measurements showed a non-monotonic trend, initially increasing before declining. Correlation analysis with previous SEM observations confirmed that membrane porosity increased proportionally with PVP content, directly influencing the mechanical behavior of the composite structures. At 5 wt.% PVP, maximum stress values of 0.73 MPa, 1.46 MPa, and 1.91 MPa were recorded for PCM-1, PCM-2, and PCM-3, respectively. This mechanical response can be attributed to PAN’s primary role as the load-bearing component within the composite structure. The enhanced tensile properties observed with increasing PAN content were complemented by PVDF’s mechanical contribution at lower porosity levels, resulting in an inverse relationship between stress performance and porosity. The strain characteristics were predominantly governed by the porous PVDF structure, with optimal strain values of 39.28%, 33.92%, and 19.62% achieved for PCM-1, PCM-2, and PCM-3, respectively, at 10 wt.% PVP. The morphological evolution of pore structure discontinuous micropores at low PVP concentrations to interconnected networks at higher concentrations–significantly influenced strain behavior. PVP concentrations exceeding 10 wt.% resulted in substantial deterioration of strain properties, indicating structural discontinuity and extensive PVDF fiber fracture.

Comparative analysis revealed that PCM-1 and PCM-3 exhibited opposing stress-strain characteristics, precluding simultaneous optimization of both properties. PCM-2 with 10 wt.% PVP emerged as the optimal formulation through a comprehensive evaluation of multiple critical factors. This membrane achieved an ideal balance between mechanical strength (1.46 MPa) and strain capability (33.92%) while maintaining moderate porosity that facilitated effective AgNP loading in subsequent modifications. The stability of its pore structure was further validated through residual strain analysis (Figure 4c), demonstrating consistent mechanical performance under cyclic loading. Moreover, the 10 wt.% PVP content provided sufficient pore formation without compromising the membrane’s structural continuity, ensuring optimal mechanical integrity. This comprehensive evaluation of mechanical properties, structural stability, and potential functionality led to the selection of PCM-2 containing 10 wt.% PVP for subsequent investigations.

FTIR spectroscopic analysis was conducted to confirm the complete extraction of PVP from the selected membrane formulation. Figure 4d shows the overlaid FTIR spectra of the PVDF/PVP/PAN composite before and after PVP dissolution, with magnified insets highlighting the regions of characteristic peaks. Before PVP dissolution, the spectrum exhibits characteristic PVP absorption bands at 1650 cm^−1^ (C=O stretching vibration) and 1420 cm^−1^ (C-H bending vibration). After PVP dissolution, these characteristic peaks disappear completely, while the distinctive absorption bands of PVDF remain unchanged, including the C-F bond stretching vibration at 840 cm^−1^, C-F bond vibration at 1170 cm^−1^, and CH_2_ group bending vibration at 1400 cm^−1^. Additionally, the characteristic absorption band at 2240 cm^−1^, attributed to the C≡N stretching vibration, confirms the presence of PAN in the composite structure. The complete disappearance of PVP characteristic peaks substantiates the successful removal of PVP during the extraction process, validating the preparation of the porous membrane structure.

### 3.2. Characteristics of Conductive Porous PVDF/PAN Composite Fibrous Membrane

The influence of silver nitrate concentration on the properties of conductive porous PVDF/PAN composite fibrous membranes was systematically investigated through comprehensive characterization techniques, encompassing microscopic morphological analysis and conductivity measurements. The optimization of reaction parameters for silver nanoparticle formation was achieved through detailed experimental studies. Scanning Electron Microscopy (SEM), analysis enabled direct visualization of the in-situ reduction process from silver ions (Ag^+^) to AgNPs and their subsequent integration within the composite membrane structure. This high-resolution imaging technique provided critical insights into the morphological evolution and spatial distribution of AgNPs across the membrane surface, facilitating a detailed mechanistic understanding of the reduction process and subsequent nanoparticle incorporation.

**Figure 5:**
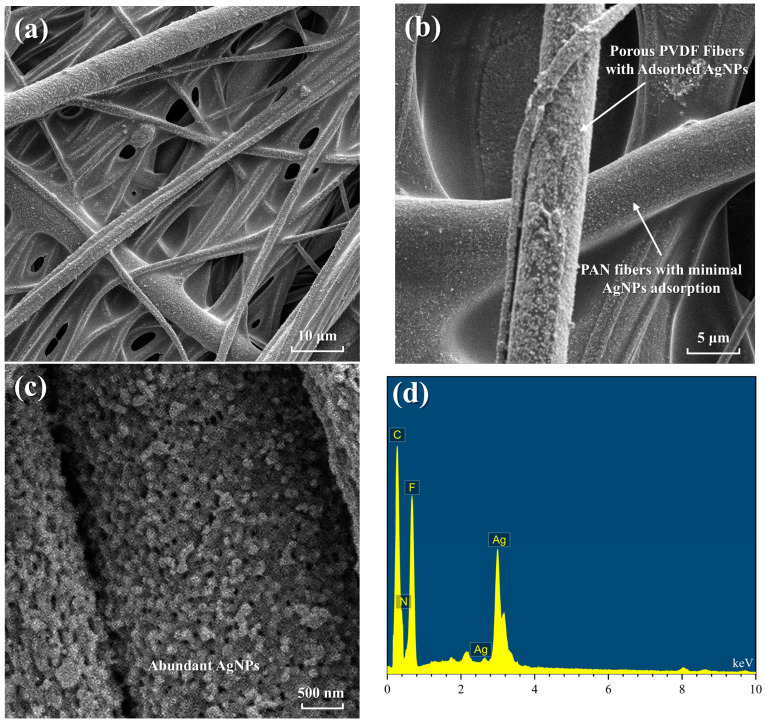
Morphological and compositional characterization of PVDF/PAN composite membrane: (**a**) interwoven network structure, (**b**) AgNPs distribution on different fibers, (**c**) abundant AgNPs on PVDF fiber surface, and (**d**) EDX spectrum of the composite.

The initial SEM analysis (Figure 5a) reveals the intricate architecture of the PVDF/PAN composite fibrous membrane, characterized by an extensively interwoven network structure. This complex fibrous matrix, formed by the interlacing of PVDF and PAN fibers, provides a substantial surface area for silver ion adsorption. The structural integration of chemically stable PVDF fibers with mechanically robust PAN fibers establishes an optimal foundation for subsequent silver ion reduction and nanoparticle distribution. Higher magnification imaging (Figure 5b) reveals a stark contrast in AgNP distribution between fiber types: while PVDF fibers exhibit significant AgNP adsorption, PAN fibers demonstrate minimal particle attachment. This distinct difference stems from the inherent surface characteristics of each fiber type. The smooth, non-porous surface of PAN fibers provides limited anchoring sites for AgNP attachment, whereas the porous PVDF structure, created through PVP extraction, offers numerous attachment points for particle adhesion. Further magnified examination (Figure 5c) reveals both individual AgNPs and some aggregate clusters on PVDF fiber surfaces. Quantitative analysis of the high-magnification SEM image shows that individual AgNPs predominantly exhibit spherical morphology with diameters ranging from 80 to 160 nm, while aggregate clusters typically measure 300–500 nm in size. This particle size distribution and aggregation behavior directly influences the formation of conductive pathways throughout the membrane structure, with optimal conductivity achieved when particle aggregation is minimized.

Additionally, Energy Dispersive X-ray (EDX) spectroscopy (Figure 5d) confirms the chemical composition and successful incorporation of AgNPs. The spectrum exhibits characteristic peaks for C and F from PVDF, N from PAN, and strong Ag peaks, demonstrating effective adhesion of silver nanoparticles to the fiber surface. The significant intensity of Ag peaks indicates substantial and uniform AgNP loading across the fibrous network, validating our in-situ reduction approach for achieving stable particle attachment.

**Figure 6:**
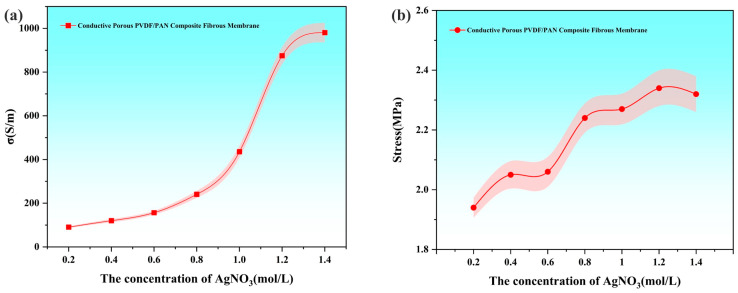
Effect of AgNO_3_ concentration on the electrical (**a**) conductivity and (**b**) stress of conductive porous PVDF/PAN composite fibrous membranes.

Figure 6 depicts the relationship between AgNO_3_ concentration and the resultant electrical conductivity and mechanical stress of the conductive porous PVDF/PAN composite fibrous membranes. The electrical conductivity demonstrates a biphasic response to increasing AgNO_3_ concentration, characterized by initial growth followed by plateau formation, while mechanical stress exhibits a general ascending trend.

In the lower AgNO_3_ concentration range (0.2–0.6 mol/L), electrical conductivity increases progressively due to enhanced AgNP deposition on the membrane surface, facilitating the formation of conductive pathways. A significant conductivity surge occurs between 0.8 and 1.2 mol/L, reaching 874.93 S/m at 1.2 mol/L, followed by a modest increase to 980.57 S/m at 1.4 mol/L. This decelerated growth at higher concentrations stems from AgNP aggregation, resulting in non-uniform distribution and elevated inter-particle contact resistance, thereby limiting further conductivity enhancement.

Mechanical stress analysis reveals the structural impact of AgNPs incorporation across varying AgNO_3_ concentrations. Within the 0.2–1.2 mol/L range, increasing AgNP content strengthens the fibrous matrix, elevating stress values from 1.94 MPa to 2.34 MPa. Beyond 1.2 mol/L, AgNPs adsorption approaches saturation, with excessive particle loading potentially compromising PVDF fiber continuity, manifested as a slight stress reduction to 2.32 MPa. Notably, the overall stress enhancement compared to pristine PVDF/PAN membranes can be attributed to improved inter-fiber bonding through AgNP deposition.

The optimal AgNO_3_ concentration was determined to be 1.2 mol/L based on comprehensive performance evaluation. At this concentration, moderate and uniform AgNP distribution ensures robust fiber-particle interactions, resulting in superior conductivity (874.93 S/m) and mechanical integrity (2.34 MPa). This optimization balances technical performance with economic considerations, as higher AgNO_3_ concentrations yield diminishing returns while increasing production costs. The selected concentration represents an optimal compromise between performance enhancement and economic feasibility.

### 3.3. Basic Sensing Performance Analysis of Conductive Porous PVDF/PAN Composite Fibrous Membrane

**Figure 7:**
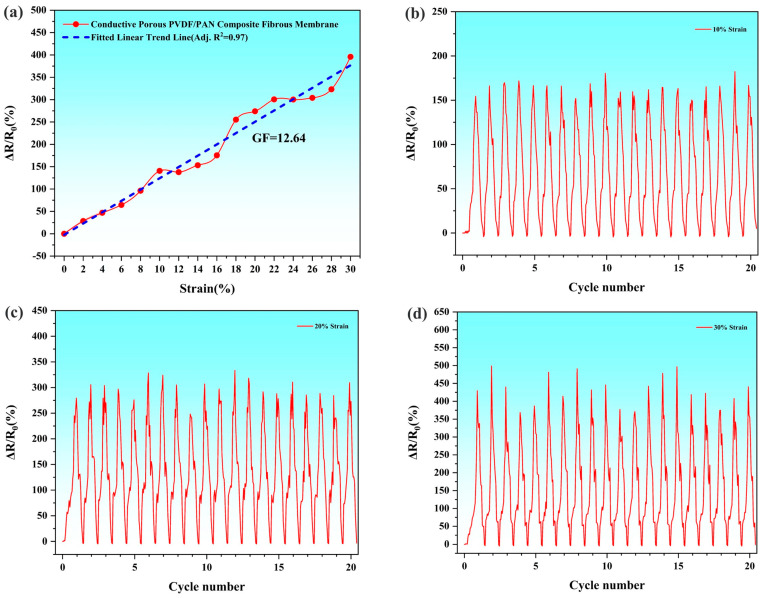
Tensile test results of the Conductive Porous PVDF/PAN Composite Fibrous Membrane: (**a**) linearity of the Conductive Porous PVDF/PAN Composite Fibrous Membrane, (**b**) linearity change at 10% strain, (**c**) linearity change at 20% strain, (**d**) linearity change at 30% strain.

Figure 7a presents the relative resistance change (Δ*R*/*R*_0_) response curve of the conductive porous PVDF/PAN composite fibrous membrane. The membrane, fabricated using 1.2 mol/L AgNO_3_ solution, demonstrates a strain sensitivity (gauge factor) of 12.64 within the strain range of 0–30%. Building upon the established principle demonstrated by Zhao et al. [33] and Cao et al. [41], where conductive materials embedded in fibrous membranes enable effective strain sensing, our study utilizes AgNPs for their superior electrical conductivity. The metallic nature of silver nanoparticles, combined with their uniform distribution throughout the fibrous network, facilitates enhanced electron transfer efficiency and, consequently, improved sensing performance.

The strain-dependent behavior of Δ*R*/*R*_0_ demonstrates distinct mechanistic transitions across different strain regimes. At low strain levels, the conductive network maintains its integrity, with AgNPs retaining sufficient inter-particle contact, resulting in minimal resistance fluctuations. As strain increases, the progressive separation of AgNPs leads to partial disruption of conductive pathways, manifesting as enhanced Δ*R*/*R*_0_ fluctuations. This phenomenon intensifies with increasing strain due to the gradual deterioration of inter-particle connections and the consequent disruption of conductive networks. Under elevated strain conditions, structural changes become more pronounced, involving potential PVDF fiber deformation and partial detachment of AgNPs from fiber surfaces, leading to significant disruption of the conductive network and marked resistance increases. The composite’s mechanical response is significantly influenced by its dual-fiber architecture, where the PAN fiber framework serves as a structural scaffold, providing essential mechanical support and strain distribution throughout the membrane. This supporting network helps maintain the structural integrity of the PVDF/AgNPs conductive system, moderating resistance changes under applied strain. The mechanical synergy between PAN and PVDF components contributes to the overall stability of the conductive network, resulting in more controlled Δ*R*/*R*_0_ variations within the measured strain range, while the PAN fiber skeleton effectively buffers external loads and preserves the composite membrane’s structural integrity.

Further analysis of the strain-sensing capabilities focused on the Δ*R*/*R*_0_ response at discrete strain levels of 10%, 20%, and 30%. Figure 7b–d demonstrates distinct linear response characteristics at each strain magnitude. The composite fibrous membrane exhibits maximum linearity ranges of 150.24–182.4% at 10% strain, 248.4–333.5% at 20% strain, and 368.9–498.95% at 30% strain. These well-defined, strain-specific linear responses indicate robust sensing performance across multiple deformation regimes. The systematic increase in linearity with strain magnitude, coupled with consistent response patterns during cyclic loading, confirms the membrane’s capability for reliable strain detection and signal transduction. It is worth noting that in multiple strain cycle tests, the Δ*R*/*R*_0_ curves of the composite fibrous membrane can overlap well without obvious drift phenomena. This demonstrates that the attachment of AgNPs to the PVDF fiber surface is firm, without apparent shedding, and the conductive layer structure is stable. Even under the action of large strains, the composite fibrous membrane can still maintain stable sensing performance, reflecting good structural integrity and mechanical durability. The stable conductive layer structure and excellent mechanical properties are key factors for the composite fibrous membrane to achieve reliable strain sensing.

The conductive porous PVDF/PAN composite fibrous membrane demonstrates significant potential for strain sensing applications, with performance characteristics suitable for flexible sensors and wearable devices. The synergistic design combines uniformly distributed AgNPs on PVDF fiber surfaces with a supporting PAN fiber framework, resulting in reproducible sensing characteristics. Systematic investigation of strain-dependent resistance responses reveals distinct linear behavior across multiple deformation regimes, enabling precise signal transduction. The structural stability of the conductive network, coupled with robust mechanical properties, establishes a reliable platform for practical sensing applications. These findings advance the fundamental understanding of composite fibrous membrane design principles and contribute to the development of next-generation flexible strain sensors. The demonstrated performance metrics position this material system as a viable candidate for human motion monitoring applications.

### 3.4. Human Sensing Performance Analysis of Conductive Porous PVDF/PAN Composite Fibrous Membrane

**Figure 8:**
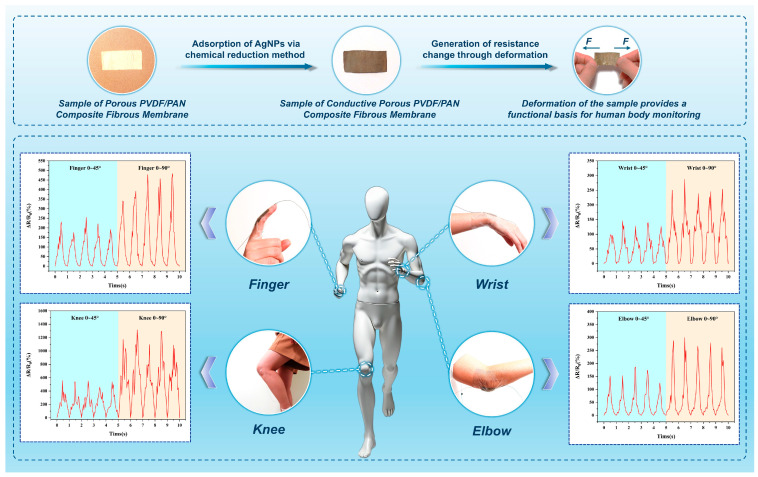
Physical appearance, sensing principle and human motion sensing performance of the conductive porous PVDF/PAN composite fibrous membrane.

The practical sensing capabilities of the conductive porous PVDF/PAN composite fibrous membrane were evaluated through human motion detection experiments. The membrane samples used for human motion detection exhibited dimensions of approximately 50 mm × 20 mm × 0.5 mm (length × width × thickness). The porous PVDF/PAN composite fibrous membrane initially appears white due to its interconnected fiber network structure. After the adsorption-reduction process, the membrane transforms to a characteristic metallic gray-black appearance with uniformly distributed AgNPs throughout the fibrous network. The sensing mechanism relies on resistance changes generated when mechanical deformation alters the spacing between AgNPs in the conductive network. The membrane sensors were strategically positioned on four anatomical locations (fingers, wrists, elbows, and knees) using transparent polyethylene (PE) tape to monitor strain signals during joint articulation (Figure 8); the membrane maintains consistent physical appearance and structural integrity when attached to different joint locations, demonstrating its adaptability to various anatomical surfaces. The PE tape, characterized by its adhesive thin-film structure and exceptional strain tolerance (significantly exceeding that of the fabricated membrane), served dual functions: protecting the conductive composite membrane from mechanical damage while providing robust adhesion to the skin surface during dynamic movements. This configuration ensured stable sensor attachment and reliable signal acquisition during large amplitude movements without mechanical failure. Controlled measurements were conducted at two distinct angular positions (45° and 90°) for each joint movement, with corresponding relative resistance changes (Δ*R*/*R*_0_) recorded throughout the motion cycles.

The sensor demonstrated distinct and reproducible response patterns across all tested anatomical locations. Analysis of the relative resistance changes (Δ*R*/*R*_0_) revealed location-specific sensing characteristics correlating with joint mobility and underlying tissue composition. The wrist, exhibiting limited flexibility and range of motion, generated the lowest Δ*R*/*R*_0_ values: 98.90–146.28% for 0–45° flexion and 238.61–287.25% for 0–90° flexion. Slightly higher responses were observed at the elbow, with Δ*R*/*R*_0_ ranges of 124.65–187.06% (0–45°) and 260.88–300.24% (0–90°), attributed to the abundant muscular tissue that moderated membrane deformation during joint articulation. Finger movements, characterized by enhanced degrees of freedom, produced substantially higher Δ*R*/*R*_0_ values: 177.01–256.40% for 0–45° flexion and 341.61–483.20% for 0–90° flexion. The knee joint exhibited the maximum response magnitude, with Δ*R*/*R*_0_ ranges of 459.60–557.84% (0–45°) and 1095.59–1316.48% (0–90°), owing to its extensive range of motion and relatively sparse muscle tissue coverage. Notably, knee measurements displayed pronounced peak fluctuations compared to other locations, manifesting as increased sawtooth patterns in the response waveform. This phenomenon likely resulted from localized structural modifications in both PAN and PVDF components under extreme strain conditions. While motion detection remained feasible, these observations suggest avoiding applications involving large-amplitude knee deformations to maintain optimal sensor performance.

The experimental results illustrated in Figure 8 demonstrate the membrane’s exceptional motion-sensing capabilities across diverse anatomical locations, including digital, carpal, cubital, and patellar articulations, as well as laryngeal vibrations. The conductive porous PVDF/PAN composite fibrous membrane exhibited remarkable sensitivity and signal fidelity in detecting both large amplitude joint movements and subtle physiological motions. This comprehensive characterization of the electrospun conductive PVDF-based fibrous membrane system establishes a robust foundation for advancing functional fiber materials in human-machine interface applications. The findings not only validate the membrane’s practical utility but also contribute significantly to the expanding paradigm of wearable sensing technologies.

This investigation demonstrates the successful development of a conductive porous PVDF/PAN composite fibrous membrane with superior human motion detection capabilities. The membrane exhibits precise strain-dependent resistance modulation (Δ*R*/*R*_0_) across various joint articulations. The exceptional sensing performance stems from the synergistic combination of homogeneously distributed AgNPs across the PVDF fiber surface and the robust mechanical support provided by the PAN fiber framework. Systematic characterization of the strain-resistance relationship revealed high linearity across distinct deformation ranges, enabling accurate signal transduction and reliable motion detection. The membrane’s structural integrity, characterized by a stable conductive network and enhanced mechanical properties, establishes its viability for practical implementations in human motion monitoring systems. These findings not only validate the membrane’s functionality but also suggest broader applications in wearable electronics and human-machine interface technologies.

Additionally, the response time characteristics of the membrane sensors were systematically evaluated across different joint movements. The measurements revealed rapid response capabilities, with response times ranging from 85 to 110 ms depending on the type and scale of joint movement. Finger movements exhibited the fastest response (~85 ms), followed by wrist (~90 ms) and elbow movements (~95 ms), while knee articulation showed slightly longer response times (~110 ms) due to the larger deformation involved. These response times indicate the membrane’s suitability for real-time human motion monitoring applications, with performance metrics comparable to other reported wearable strain sensors.

## 4. Conclusions

This investigation successfully demonstrated the development and characterization of conductive porous PVDF/PAN composite fibrous membranes with embedded AgNPs through an innovative fabrication approach. The systematic optimization of processing parameters and materials composition yielded several significant findings:

The conjugate electrospinning of PVDF/PAN, combined with selective PVP dissolution and in-situ AgNP reduction, resulted in a hierarchical porous structure with enhanced electrical and mechanical properties. The optimal PVP content of 10 wt.% and AgNO_3_ concentration of 1.2 mol/L produced membranes exhibiting superior electrical conductivity (874.93 S/m) and mechanical strength (2.34 MPa).

The membrane demonstrated distinct location-specific sensing capabilities during human motion detection. For moderate-range movements, the relative resistance changes (ΔR/R0) exhibited systematic and stable responses: wrist movements generated ΔR/R0 values of 98.90–146.28% for 0–45° flexion and 238.61–287.25% for 0–90° flexion; elbow movements produced ΔR/R0 ranges of 124.65–187.06% (0–45°) and 260.88–300.24% (0–90°); and finger movements showed excellent sensitivity with ΔR/R0 values of 177.01–256.40% (0–45°) and 341.61–483.20% (0–90°). While knee movements exhibited the highest response magnitude (ΔR/R0: 459.60–557.84% for 0–45° and 1095.59–1316.48% for 0–90°), the pronounced signal fluctuations and sawtooth patterns observed suggest that the sensor is better suited for applications involving moderate joint movements rather than extreme knee deformations.

The systematic correlation between joint mobility and sensing response, particularly in moderate-range motions, demonstrates the membrane’s capability for precise and stable motion detection. The observed performance characteristics provide valuable insights for optimizing sensor design in specific wearable applications, with particular emphasis on maintaining signal stability and reliability within appropriate deformation ranges. Compared to conventional conductive fiber membrane fabrication methods such as carbon nanotube deposition and metal salt oxidant attachment, which often face conductivity-mechanical property trade-offs [42,43], our conjugate electrospinning approach demonstrates superior balanced performance, achieving both high conductivity (874.93 S/m) and excellent mechanical strength while maintaining stable strain sensing capabilities. The unique combination of PVDF’s porous structure and PAN’s mechanical support overcomes the typical limitations of single-component systems, offering a promising pathway for next-generation flexible sensors.

This study demonstrates the potential of conjugate electrospun PVDF/PAN membranes in flexible sensing applications. The findings suggest several directions for future research, including the optimization of fiber diameter ratios and porosity control to enhance sensing stability under moderate strain conditions. The current PVDF/PAN system could potentially be modified through the incorporation of additional functional nanoparticles to achieve multi-modal sensing capabilities, such as temperature sensing or electromagnetic shielding while maintaining its motion-sensing performance within appropriate strain ranges. Process optimization for industrial-scale manufacturing presents another critical avenue for development, particularly through multi-nozzle electrospinning systems that could enable scaled production while maintaining performance consistency. Regarding practical applications, the membrane system shows considerable promise for integration into wearable healthcare monitoring devices, smart textiles with embedded sensing capabilities, rehabilitation monitoring systems, and human-machine interface technologies. These research directions collectively aim to bridge the gap between laboratory development and practical implementation, advancing the field of flexible and wearable sensing technologies. The developed fabrication methodology presents opportunities for scale-up production through multi-nozzle electrospinning systems, which may facilitate the practical implementation of these sensors in wearable healthcare monitoring devices and smart textiles.

## Data Availability

The original contributions presented in this study are included in the article. Further inquiries can be directed to the corresponding authors.
